# Identifying Network Public Opinion Leaders Based on Markov Logic Networks

**DOI:** 10.1155/2014/268592

**Published:** 2014-04-01

**Authors:** Weizhe Zhang, Xiaoqiang Li, Hui He, Xing Wang

**Affiliations:** School of Computer Science and Technology, Harbin Institute of Technology, Harbin 150001, China

## Abstract

Public opinion emergencies have important effect on social activities. Recognition of special communities like opinion leaders can contribute to a comprehensive understanding of the development trend of public opinion. In this paper, a network opinion leader recognition method based on relational data was put forward, and an opinion leader recognition system integrating public opinion data acquisition module, data characteristic selection, and fusion module as well as opinion leader discovery module based on Markov Logic Networks was designed. The designed opinion leader recognition system not only can overcome the incomplete data acquisition and isolated task of traditional methods, but also can recognize opinion leaders comprehensively with considerations to multiple problems by using the relational model. Experimental results demonstrated that, compared with the traditional methods, the proposed method can provide a more accurate opinion leader recognition and has good noise immunity.

## 1. Introduction

As the Internet enters into the We-media era, every individual can be a message sender. However, public opinion emergencies may affect the social activities significantly due to the mixed netizen qualities. To avoid adverse effect, it is necessary to have a comprehensive understanding of the development trend of public opinion and recognize special communities like opinion leaders.

The earliest domestic and foreign researches on the discovery of network opinion leaders focused on using the opinion leader theory in the traditional social sciences and research method transplantation directly to recognize the internet leaders but failed to achieve ideal results. They often determined community opinion leaders based on the quantitative data analysis. These methods [1], regardless of the characteristics of objective fact, only depend on logic reasoning and could not represent the public opinion transmission characteristics on the new media of network. Recently, scholars began to explore the difference between network environment and offline practical society through quantitative measurement. The accuracy of the leader discovery model [2–5] witnessed a continuous improvement from the clustering analysis of statistical properties [6] to social network analysis based on complex network [7–9] and to the semantic analysis of text content.

### 1.1. Attributes of Public Opinion Participants

Behaviors of every netizen involved in the public opinion transmission are described by inherent attribute, content attribute, and social network attribute.

Inherent attribute refers to the independent attributes of participants from the concerning public opinion events such as career, position, internet age, logins, community credits, fans population, and concerns.

Content attribute refers to the behavioral description of the participants in a certain public opinion event including posts, replies, comments received, reposts, number of mentions, number of words, and emotional tendency.

Social network attribute refers to the mutual relationship of participants in the network mainly including fans and concerns of the participants.

### 1.2. Shortcomings of Existing Research Methods

Existing network opinion leader discovery is based on the recognition model involving only one or two attributes of participants. No network opinion leader recognition involving all three attributes of participants has been reported yet. This will affect the accuracy of the opinion leader recognition method.

Existing network opinion leader discovery views the attributes of participants as independent and identically distributed (IID) data. In the theory of probability statistics, mutually independent sequence of variables or other random variables that have the same probability distribution is called IID. However, attribute data of participants are relational data. Different attributes of participants are mutually correlated instead of being independent from each other. For example, fans' population of inherent attribute often is proportional to comments received of content attribute; participants enjoying high attentions from opinion leader are more likely to be the opinion leader. The incomprehensive understanding of such relations will make some opinion leaders remain unidentified. Furthermore, existing network opinion leader discovery has no modeling solution to the relational data.

As a result, simultaneous application of all three attributes and the exploration of relationships in public opinion data can improve the performance of network opinion leader recognition method.

## 2. Markov Logic Networks

Markov Logic Networks refer to a learning method of statistical relation that is combining the Markov network and first-order logic together. It was proposed by Singla and Domingos [10] in 2004 and then improved by Domingos and his students.

Markov network [11] is a joint distribution model of random variable set (*X* = (*x*_1_, *x*_2_,…, *x*_*n*_)). It is composed of an undirected graph (G) and a potential function (Φ_*k*_) set. Every random variable occupies a node of the graph and each group has a potential function in the model. Potential function is a nonnegative real function, which represents the state of the corresponding group. The joint distribution of Markov network is as follows:

(1) $$\begin{array}{c} P\left(X=x\right)=\frac{1}{Z}\prod_{k}\Phi_{k}\left(x_{\left\{k\right\}}\right), \end{array}$$

where *x*_{*k*}_ is the state of random variables in the group and *Z* is partition function (state sum) that is defined as ∑_*x*∈*X*_∏_*k*_Φ_*k*_(*x*_{*k*}_). Weight all characteristic values of potential-use states of each group in the Markov network. Then sum them and calculate the exponentiation. Finally, a log-linear model can be gained as follows:

(2) $$\begin{array}{c} P\left(X=x\right)=\frac{1}{Z}\exp\left(\sum_{j}\omega_{j}f_{j}\left(x\right)\right). \end{array}$$

Characteristic function can be any real function of state. In this paper, characteristic function refers to the dual characteristics value ({0,1}). Equation (1) is the most direct expression of potentials, in which every possible state of each group has a corresponding characteristic value and a weight. Equation (1) is related to the power of groups. However, the amount of characteristic values can be reduced freely by some methods (e.g., logic function of state), thus enabling the characteristic function to provide a simpler expression under large number of groups compared with the potential functions. This is the principle of the Markov Logic Networks.

Markov Logic Networks are a first-order logic knowledge base where every code has a weight. This first-order logic knowledge base can be viewed as the template of Markov Logic Networks. Viewed from probability, the Markov Logic Networks provide a simple language to define large Markov network as well as a flexible and modularized integration with abundant knowledge. Viewed from the first-order logic, the Markov Logic Networks provide a sound processing to knowledge base with uncertainties, defects, and even contradictories, thus decreasing the vulnerability.

Take the data set of Skyline for example. The simplest situation is as follows: suppose the knowledge base only contains the formula *F*_1_ (weight = 1.5), and the corresponding MLN of the knowledge base is {(*F*_1_, 1.5)}:

(3) F1:PostHasWord(y,x)   ⟶(BadWord(x)⟶BadPost(y)),

where *x* and *y* are individual variables; BadWord(*x*), BadPost(*y*), and PostHasWord(*y*, *x*) are predicates, representing whether *x* has bad word, *y* is a bad post, and *y* contains *x*. *F*_1_ means that if *y* has the bad word*x*, *y* can be deduced as a bad post.

**Figure 1:**
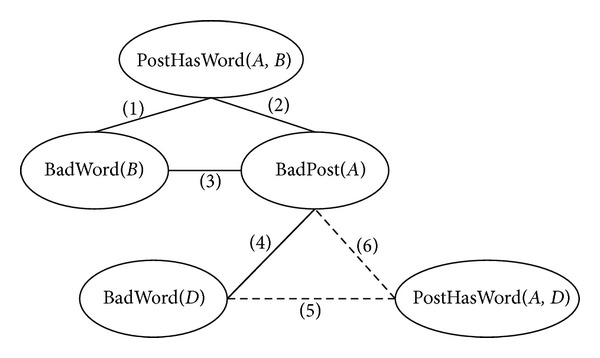
Closed Markov Logic Network.

Given an individual constant set *C* = {*A*, *B*, *D*}, a closed Markov Logic Network can be generated (Figure 1).

### 2.1. Markov Logical Reasoning Method

Markov logical reasoning is equal to the probabilistic reasoning of the complex relationship. The basic task of reasoning is to inference the most possible state of world *y* according to the given evidence *x* (word set).

There are two basic types of reasoning: First, we search the most possible state satisfying some evidence and probability of computer random condition. Next, lazy reasoning and relieving reasoning are compared in improving the performance of above two types of reasoning in processing more complicated relationships. The lazy reasoning only requires adopting instantiation “default” value to the deviated basic values, while relieving reasoning divides indistinctive atoms into one group and views them as an independent unit.

### 2.2. Markov Logic Networks Learning

The Markov Logic Networks learning includes structure learning and parameter learning. Structure learning is to learn the model structure (network structure of the Markov Logic Networks) from data. In structure learning, it is more difficult to learn the rules. There are two structure learning methods based on the inductive logic programming (ILP): (1) learn model structure by using CLAUDIEN (an LP system) directly; (2) learn structure of Markov Logic Networks from the relational database by combining LP and feature introduction in Markov network together. Parameter learning is committed to find the satisfying rule weight with maximum likelihood or maximum conditional likelihood of associated database. Parameter learning includes production parameter learning and discriminant parameter learning. Parameter learning is needed under known structure of Markov network to acquire weight of each rule. However, when the structure of Markov network is unknown, structure learning is needed before the parameter learning to get the network structure.

Markov Logic Networks learning includes parameter learning and structure learning. Parameter learning has production parameter learning and discriminant parameter learning as well as their corresponding formulas and approximate algorithms. Structure learning also includes top-down structure learning and bottom-up structure learning.

## 3. System Framework

**Figure 2:**
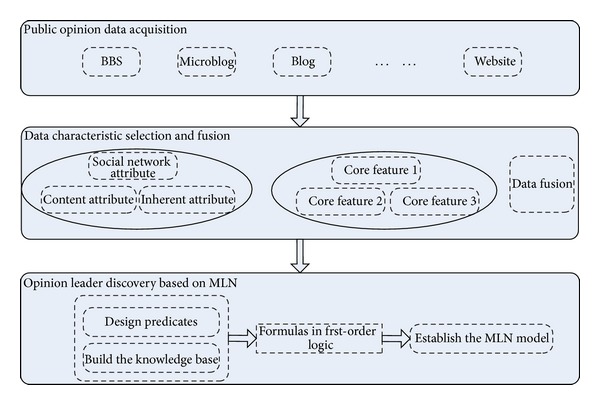
Overall structure of the network opinion leader recognition system based on the Markov Logic Networks.

The overall structure of the network opinion leader recognition system based on the Markov Logic Networks is shown in Figure 2.

The designed network opinion leader recognition system includes three modules: public opinion data acquisition module, data characteristic selection and fusion module, and opinion leader discovery module based on Markov Logic Networks. The public opinion data acquisition module is for data collection concerning specific public opinion event. Data characteristic selection and fusion module is for processing and analyzing collected data to disclose the relationship between core characteristics and attributes. The opinion leader discovery module based on Markov Logic Networks is to design predicates, build the knowledge base, and establish the MLN model according to the relationship between core characteristics and attributes.

### 3.1. Opinion Leader Discovery Module Based on Markov Logic Networks

**Figure 3:**
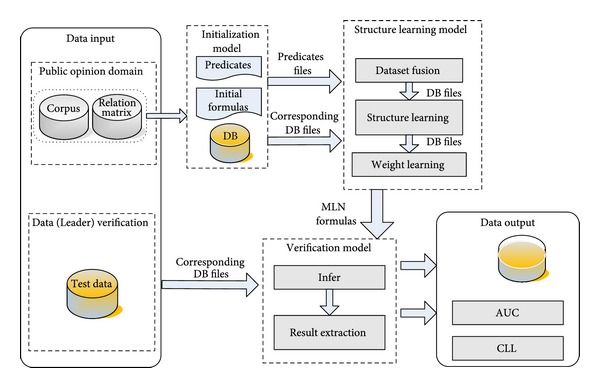
Technical route of opinion leader recognition based on Markov Logic Networks.

The technical route of opinion leader recognition based on Markov Logic Networks is presented in Figure 3. The top data input, initialization module, and structure learning module are the training module of the model, whereas the bottom data (Leader) verification, verification module, and data output are the verification module of the model.

The primary task of the training module is to design predicate. Predicate design has two stages: (1) original design of initial predicate set according to the characteristic matrix and relational knowledge gained by the data characteristic extraction and fusion module as well as personal priori knowledge; (2) repetitive reasonable adjustment of predicate design according to the learning results of the model and follow-up experimental analysis of learning results until getting satisfying experimental result.

The initialization module will convert contents in the corpus into DB according to existing predicate design. The structure learning module can conduct the structure learning through the available learnstruct program of Alchemy. Beam search is the default structure learning algorithm. Weight learning can be implemented by the available learning program. Weighted MLN clause can be gained through the structure learning and weight learning, which will be used for reasoning. In the designed system, these weighted MLN clauses are used to deduce user's identity.

In the verification module of the model, both verification data and training data are converted into DB according to the predicate design. The verification module is mainly used to deduce user's identity.

Reasoning results contain all possible predicates and their possibilities. Take the predicate of teachby(course, teacher) for instance. Course represents course and teacher represents teacher, meaning that the course is taught by the teacher. If the course is Chinese, possible teacher set is {Tom, Jack, Lily}. Then, the reasoning results may be as follows:

(4) $$\begin{array}{c} \text{teacherby}\left(\text{Chinese},\text{Tom}\right)\;0.9275\\ \text{teacherby}\left(\text{Chinese},\text{Jack}\right)\;0.7465\\ \text{teacherby}\left(\text{Chinese},\text{Lily}\right)\;0.3556. \end{array}$$

Result extraction means to select the reasoning result with highest probability as the final result. In the above case, Tom will be selected as the teacher of Chinese. The data output module will calculate AUC and CLL.

## 4. Experimental Verification

### 4.1. Recognition of Nonrelational Data Model

Netizens were classified according to the nonrelational data model provided by Weka. First of all, the original data have to be converted into ARFF. ARFF mainly includes attribute assertion and data [12, 13].

**Figure 4:**
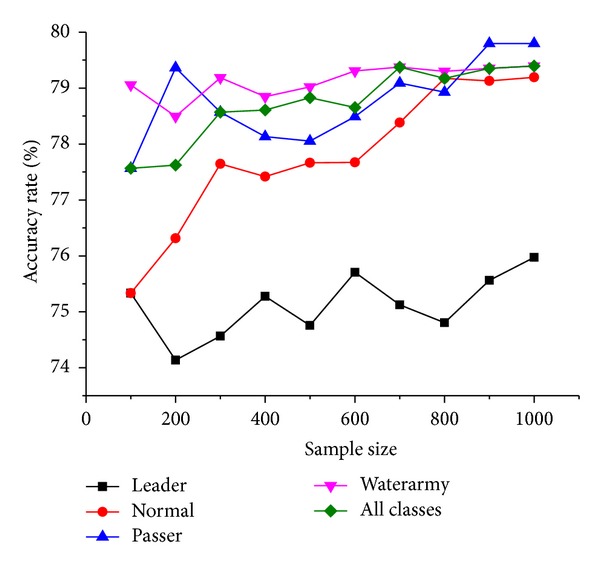
Group recognition of nonrelational data model.

We chose SVM to classify netizens involved in the “Xu-Ting Event” on the legal forum of Skyline Gossip as Leader, Normal, Passer, and Waterarmy. In our experiment, every netizen was recognized independently. Experimental results are presented in Figure 4.

It can be seen from Figure 4 that the nonrelational data model achieved a recognition accuracy of about 77%, which presented a continuous growth as the sample size increases.

### 4.2. Recognition of Relational Data Model

Firstly, we have to design predicates. Our designed predicates according to the characteristic selection and personal priori knowledge are listed in Tables 1, 2, and 3, including three classes (social network attribute, content attribute, and inherent attribute). These three classes of attribute are used to describe different network behaviors and individual characteristics of netizens.

After the predicate design, we have to convert original data into DB.

Next, we have to implement structure learning and weight learning. The input MLN file (predicate statements) for structure learning is shown as Box 1.

And the structure learning results are shown as Box 2.

The input MLN file (predicate statements and design statements) for weight learning is shown as Box 3.

The design statements are recognition results of four groups.

The weight learning results are represented as Box 4.

Table 4 lists some learned valuable clauses such as the following:

 4.27395 !reply(a1,a2) v act(a2,Leader) v !act(a1,Leader).

This clause means that if a1 is the leader and a1 replies a2, then a2 is a leader too. This is true in real life.

The learned valuable clauses were selected for reasoning of the test set. According to the experimental results (shown as Box 5), Person15, Person30, Person21, Person55, and Person51 were identified as Passer, Normal, Passer, Leader, and Passer, respectively. Four were recognized accurately and 1 was recognized wrongly.

The recognition accuracy comparison results of relational data model and nonrelational data model to different events are listed in Table 5, finding that the relational data model has higher recognition accuracy compared with the nonrelational data model.

## 5. Conclusions

This paper firstly summarizes and evaluates the shortcomings of existing opinion leader recognition method, describes the advantages of Markov Logic Networks in opinion leader recognition, and summarizes the associated theories of Markov Logic Networks including basic concepts as well as theoretical models (reasoning and learning). The Markov Logic Networks combine the probability theory and first-order logic perfectly, integrating logic/relation expressions, uncertainty processing, and learning. Secondly, this paper designs and implements a network opinion leader recognition system based on previous theories. This designed system firstly collects some public opinion data as the training set for structure learning of Markov Logic Networks and then uses the learning results to reasoning the control results of corresponding public opinion domain of the test data. The experimental results are compared and analyzed to evaluate their validity. Thirdly, this paper carries out an experimental verification, which verifies the superiority of the designed network opinion leader recognition system.

## Figures and Tables

**Box 1:**
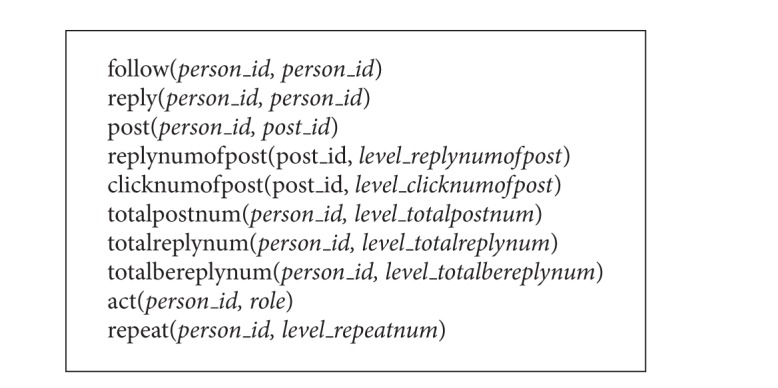


**Box 2:**
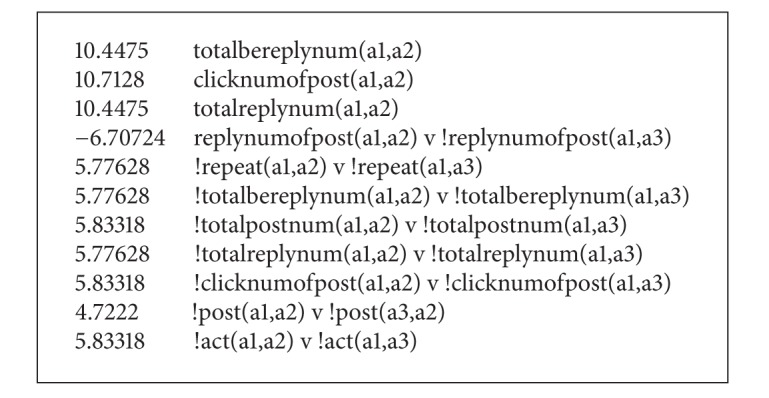


**Box 3:**
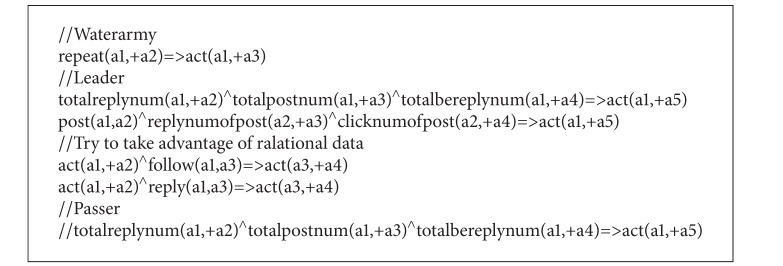


**Box 4:**
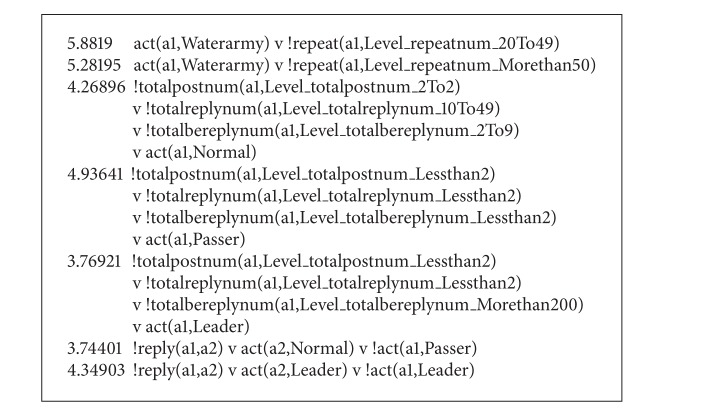


**Box 5:**
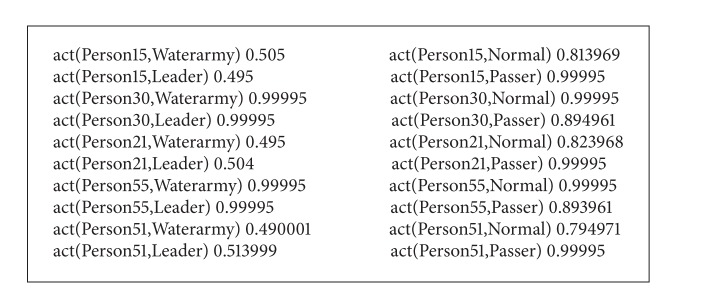


**Table 1:** Designed predicates of content attribute.

Predicate	Meaning
Post(*person_id*, *post_id*)	User who is represented by *person_id* published a post which is represented by* post_id*.

ReplyNumOfPost(*post_id*, *level_replynumofpost*)	The reply number of the post which is represented by *post_id *is *level_replynumofpost*.

ClickNumOfPost(*post_id*, *level_clicknumofpost*)	The click number of the post which is represented by *post_id* is level_clicknumofpost.

TotalPostNum(*person_id*,* level_totalpostnum*)	The post number of the user who is represented by *person_id* is *level_totalpostnum*.

TotalReplyNum(*person_id*,* level_totalreplynum*)	The reply number of the user who is represented by *person_id* is *level_totalreplynum*.

TotalBeReplyNum(*person_id*, *level_totalbereplynum*)	The number of replies to the user who is represented by *person_id* is* level_totalbereplynum*.

Correlation(*person_id*, *post_id*, *level_correlation*)	The correlation level between the user who is represented by *person_id* published content in the post which is represented by *post_id* and post topics is* level_correlation*.

Sentiment(*person_id*,* level_sentiment*)	The degree of the emotional tendencies bases on the content published by the user who is represented by *person_id* is *level_sentiment*.

**Table 2:** Designed predicates of social network attribute.

Predicates	Meaning
FansNum(*person_id*, *level_fansnum*)	The fans number of the user who is represented by *person_id* is *level_fansnum*.

FollowNum(*person_id*,* level_follownum*)	The follow number of the user who is represented by *person_id* is *level_follownum*.

Follow(*person_id*, *person_id*)	A user who is represented by the first *person_id* follows another user who is represented by the second* person_id*.

Reply(*person_id*, *person_id*, *post_id*, *num*)	In the post which is represented by the *post_id*, the number of a user who is represented by the first *person_id* reply to another user who is represented by the second *person_id* is *num*.

**Table 3:** Designed predicates of inherent attribute.

Predicate	Meaning
Gender(*person_id*, *gender*)	The gender of the user who is represented by *person_id* is* gender*.

Age(*person_id*, *level_age*)	The age of the user who is represented by *person_id* is *level_age*.

NetworkAge(people,* level_networkage*)	The network age of the user who is represented by *person_id* is *level_networkage*.

LogNum(*person_id*,* level_lognum*)	The login number of the user who is represented by *person_id* is *level_lognum*.

CommunityCredits(*person_id*, *level_communitycredits*)	The community credits of the user who is represented by *person_id* is *level_communitycredits*.

HasPosition(*person_id*)	The user who is represented by *person_id* has a communities position.

Role(*person_id*, *role*)	The role of the user who is represented by *person_id* is *role*.

**Table 4:** Valuable clauses learned from the “Xu-Ting Event”.

Weight	Formula
2.76133	!fansnum(a1,Level_fansnum_10To49) v !follownum(a1,Level_follownum_Lessthan10) v gender(a1,Male) v !networkage(a1,Level_networkage) v !lognum(a1,Level_log_num_1000To4999) v !communitycredits(a1,Level_communitycredits_1000To9999)

2.63516	gender(a1,Female) v !lognum(a1,Level_log_num_1000To4999)

3.21897	!fansnum(a1,Level_fansnum_10To49) v !follownum(a1,Level_follownum_Lessthan10) v age(a1,Level_realage_Morethan35) v !networkage(a1,Level_networkage) v !lognum(a1,Level_log_num_1000To4999) v !communitycredits(a1,Level_communitycredits_1000To9999)

3.77246	gender(a1,Female) v !lognum(a1,Level_log_num_Lessthan1000)

4.27395	!reply(a1,a2) v act(a2,Leader) v !act(a1,Leader)

5.65332	gender(a1,a2) v !age(a1,a3) v !age(a1,a4) v lognum(a1,a5) v lognum(a1,a6)

6.06442	!communitycredits(a1,a2) v !communitycredits(a1,a3) v !totalreplynum(a1,a4) v !totalreplynum(a1,a5)

6.06101	!networkage(a1,a2) v !networkage(a1,a3)

**Table 5:** Recognition accuracy comparison of relational data model and non-relational data model.

Event name	Forum	The accuracy of IID model (%)	The accuracy of relation model (%)
Xu-Ting Event	Legal Forum	79.5	82.5
Xu-Ting Event	Tianya By-talk	77.4	80.6
Three years of great Chinese famine	Discussion about the history	77.8	81.8
Three years of great Chinese famine	Tianya By-talk	76.9	80.8
